# Death of Dr. William C. Rogers

**Published:** 1860-05

**Authors:** 


— Among the recent lamented dead of our profession, we regret to record the name of Dr. Wm. C. Rogers, of Green Island, Albany Co., N. Y., who died on the 7th of April last, aged 28 years, of pneumonia.

A special meeting of the Albany County Medical Society, of which he was an honored member, was called on the 9th of April last, and resolutions appropriate to the occasion adopted.

As was said of him by one who knew him—"he was an enthusiastic and energetic worker in his profession; in his social nature, genial and happy; and to his patients, no less a friend than physician."

The pages of our journal have frequently been enriched by his contributions, and his recent death invests with peculiar interest the valuable paper by him which our present number contains, the manuscript of which reached us but a day or two before the announcement of its author's death.

